# Mapping the global research landscape on psoriasis and the gut microbiota: visualization and bibliometric analysis

**DOI:** 10.3389/fcimb.2025.1531355

**Published:** 2025-04-25

**Authors:** Yue-Min Zou, Man-Ning Wu, Xiangnan Zhou, Yan-Ping Bai

**Affiliations:** ^1^ Beijing University of Chinese Medicine, Beijing, China; ^2^ National Center for Integrative Medicine, China-Japan Friendship Hospital, Beijing, China; ^3^ Department of Dermatology, China-Japan Friendship Hospital, National Center for Integrative Chinese and Western Medicine, Beijing, China

**Keywords:** gut microbiota, psoriasis, bibliometric analysis, pathogenesis, research landscape

## Abstract

**Background:**

Psoriasis is a common chronic inflammatory skin disease with a complex pathogenesis. Recently, the role of gut microbiota in psoriasis has attracted increasing attention. A systematic bibliometric analysis of relevant literature is necessary to understand better the current state and development trends in this field.

**Materials and methods:**

The Web of Science Core Collection database was searched for literature indexed from 2004 to October 15, 2024. Bibliometric analysis was conducted using Bibliometrix, CiteSpace (version 6.3.R1), R 4.2.2 with the Bibliometrix package, Scimago Graphica 1.0.45, and VOSviewer (version 1.6.20.0) to visualize publication types, years, authors, countries, institutions, journal sources, references, and keywords.

**Results:**

The development of psoriasis and gut microbiota research can be divided into two phases: slow growth (2004–2014) and rapid development (2014–2024). Lidia Rudnicka is the most active and influential author. China produced the highest number of publications, followed by the United States, which had the highest number of citations per article. The International Journal of Molecular Sciences published the most articles. In contrast, articles in the Journal of Investigative Dermatology, British Journal of Dermatology, and Journal of Allergy and Clinical Immunology were cited over 1,000 times. Keyword and co-citation analyses identified evolving research hotspots. Early studies focused on the association between gut microbiota and comorbid inflammatory diseases. Recent research has delved into specific mechanisms, such as disruption of gut barrier function, short-chain fatty acid metabolism alterations, impaired regulatory T-cell function, and excessive activation of Th17 cells. These mechanisms highlight how gut dysbiosis exacerbates psoriasis patients’ systemic inflammation and skin lesions.

**Conclusion:**

The field of psoriasis and gut microbiota research is developing rapidly despite uneven research distribution. This bibliometric evaluation assesses the current state of research and provides new perspectives for understanding the complex interactions between microbes and the host. Future efforts should strengthen international collaboration to deeply explore the mechanisms of gut microbiota’s role in psoriasis, especially its potential applications in disease diagnosis and treatment.

## 1 Introduction

Psoriasis is a chronic immune-mediated inflammatory disorder with a multifactorial etiology, involving genetic susceptibility, immune system dysregulation, and environmental factors such as infections, stress, and dietary influences (Armstrong and Read, 2020; Sugumaran et al., 2024). As a systemic disease, psoriasis has been strongly linked to various comorbidities, including metabolic syndrome, hyperlipidemia, cardiovascular diseases, and depression and anxiety (Sugumaran et al., 2024). From 1990 to 2021, the incidence of psoriasis has been increasing globally, contributing significantly to the public health burden (GBD 2019 IMID Collaborators, 2023; Li et al., 2023). Recent advancements in high-throughput sequencing technologies have uncovered the crucial role of the gut microbiota in modulating inflammatory responses in psoriasis (Hidalgo-Cantabrana et al., 2019). As a result, research on gut microbiota has become one of the major areas of interest in understanding the pathogenesis of psoriasis (Yang et al., 2024).

The gut is a vital component of the immune and neuroendocrine systems, harboring trillions of microorganisms, including bacteria, yeasts, and parasites (Heintz-Buschart and Wilmes, 2018; Wolter et al., 2021). Most studies examining the relationship between gut microbiota and psoriasis have focused on the characteristics of the microbiota in psoriasis patients, the impact of dysbiosis on the pathological processes of psoriasis, and potential microbiome-targeted therapeutic strategies (Chen et al., 2024). Compared to healthy controls, psoriasis patients exhibit significant reductions in microbiota diversity and alterations in the abundance of specific microbial communities. This dysbiosis may influence host immune responses, promote inflammation, and thus contribute to the development of psoriasis (Codoñer et al., 2018; Hidalgo-Cantabrana et al., 2019; Chen et al., 2024). Furthermore, biological therapies have demonstrated substantial efficacy in improving psoriasis symptoms; however, their effects on regulating the gut microbiota remain an area for further investigation (Vižlin et al., 2024).

Despite preliminary evidence suggesting the potential role of gut microbiota in psoriasis pathogenesis, significant gaps remain, particularly in understanding the microbiota’s interaction with metabolic syndrome and its stage-specific effects in psoriasis. There is also a lack of studies investigating the gut microbiota characteristics in psoriasis patients across different geographic regions, disease stages, and types of psoriasis, which limits the generalizability and consistency of current findings.

Bibliometric analysis, through graphical visualization, offers a rapid way to understand the development trends and emerging hotspots in an academic field. To our knowledge, no bibliometric study has yet been conducted on psoriasis associated with metabolic syndrome, and our work aims to fill this gap. We performed a bibliometric analysis to describe the global trends, and contributions from countries, institutions, and authors, and explored the current state of research and emerging trends in the relationship between psoriasis and gut microbiota. This study aims to provide accurate and comprehensive information to clinicians, researchers, and policymakers, facilitate the rational allocation of public health resources, inspire future research directions, and offer valuable insights for clinical decision-making.

## 2 Method

### 2.1 Data selection

The Web of Science (WOS) database was used for the literature retrieval. The final search strategy was: TS=(“psoriasis” OR “psoriatic disease”) AND TS=(“microbiota” OR “microbiome” OR “flora” OR “microbial community”). Literature published from the establishment of the database (2003) to October 15, 2024, was considered. The document types were limited to articles and review articles, and the publication language was restricted to English. A total of 588 articles met the inclusion criteria, including 335 research articles and 254 review articles. The selected publications were exported in a plain text file format, following the “full record and cited references” format. To ensure data reliability, two authors independently conducted the retrieval using the same search strategy, and any discrepancies were cross-checked.

### 2.2 Visualization analysis

**Figure 1:**
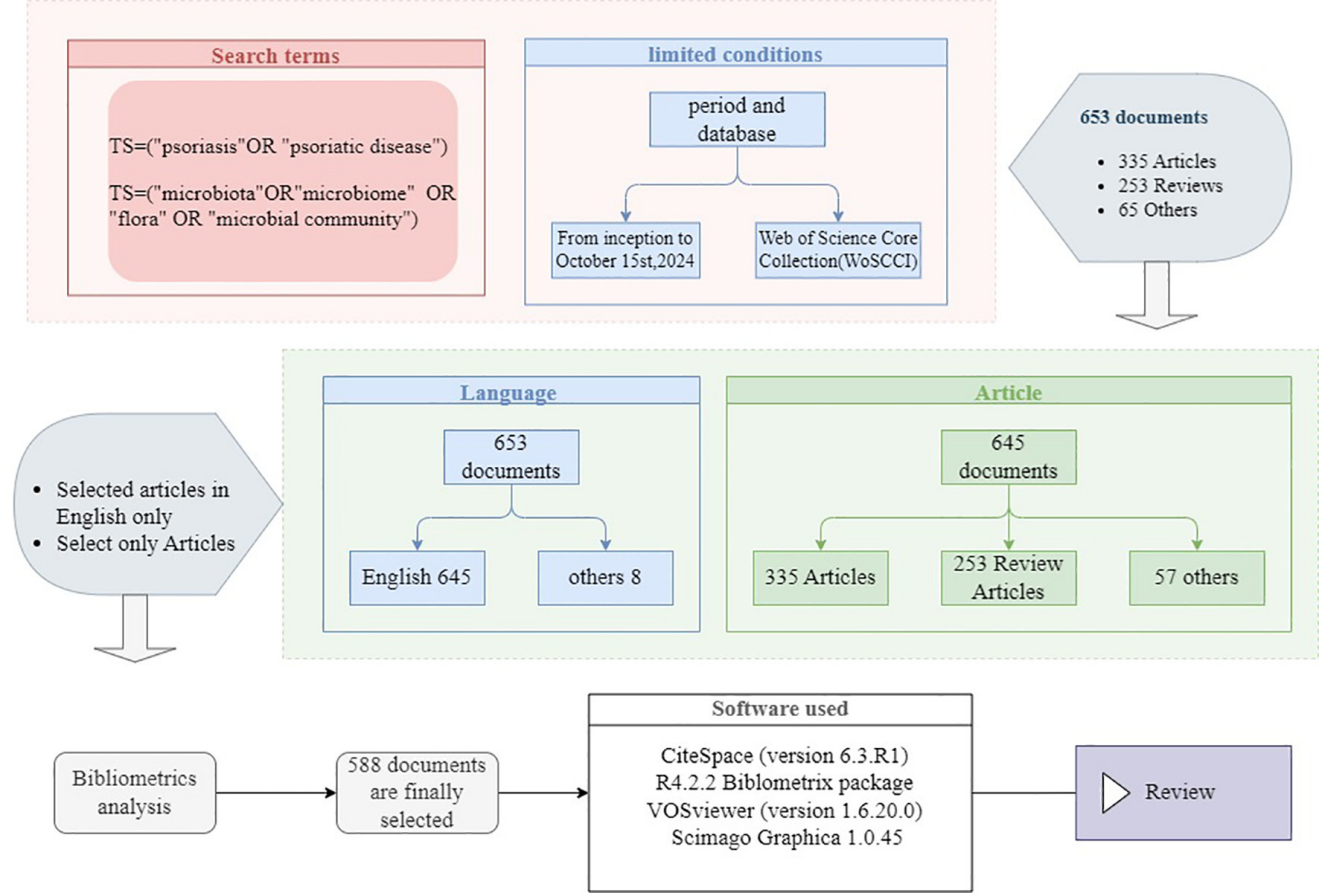
Flowchart of the bibliometric study on psoriasis and gut microbiota research.

Citation analysis and visualization of authors, journals, years, countries, keywords, research objectives, and publications were conducted using CiteSpace (version 6.3.R1), R4.2.2 Biblometrics package, Scimago Graphica 1.0.45 and VOSviewer (version 1.6.20.0). The overall workflow of the analysis is shown below (**Figure 1**).

## 3 Result

### 3.1 Publication number and citation frequency as indicators of research development

The number of publications and citation frequency are essential indicators of the development trajectory and potential growth of a research field. Between 2004 and 2014, the research output remained relatively stable, with the annual number of publications generally staying below 10. The second phase, from 2014 to 2024, marked a period of growth, with a noticeable increase in publications, particularly in 2021 when the number of publications peaked. From 2021 to 2024, the publication volume exhibited a fluctuating trend, but our study only includes data up to October 2024, meaning that publication volume and citation frequency for 2024 may continue to rise. The rise in publications can be attributed to the rapid adoption of high-throughput sequencing technologies for analyzing gut microbiota, which has made it possible to analyze unculturable bacteria, thus significantly enriching the gut microbiome database and linking microbial characteristics to specific physiological states or diseases (Quince et al., 2017). The field’s average Annual Growth Rate (AGR) is 17.91%, indicating strong potential for further research on psoriasis and the gut microbiota.

### 3.2 Research output by country and institution

This study includes 1158 institutions across 68 countries and regions, with 3471 authors contributing. **Supplementary Table 1** provides a breakdown of the top ten countries in terms of academic output. The United States leads in total citation frequency (5662) and average citations per article (59), surpassing most other countries globally. Among the top ten countries, two institutions from the United States—specifically the University of California System—emerged as the most productive research institutions, publishing 58 articles, reflecting the country’s strong scientific leadership in psoriasis and gut microbiota research. Notably, while China ranks lower than the United States and Italy in citation frequency, it has the highest number of publications, accounting for 21.1% of the total number of articles.

**Figure 2:**
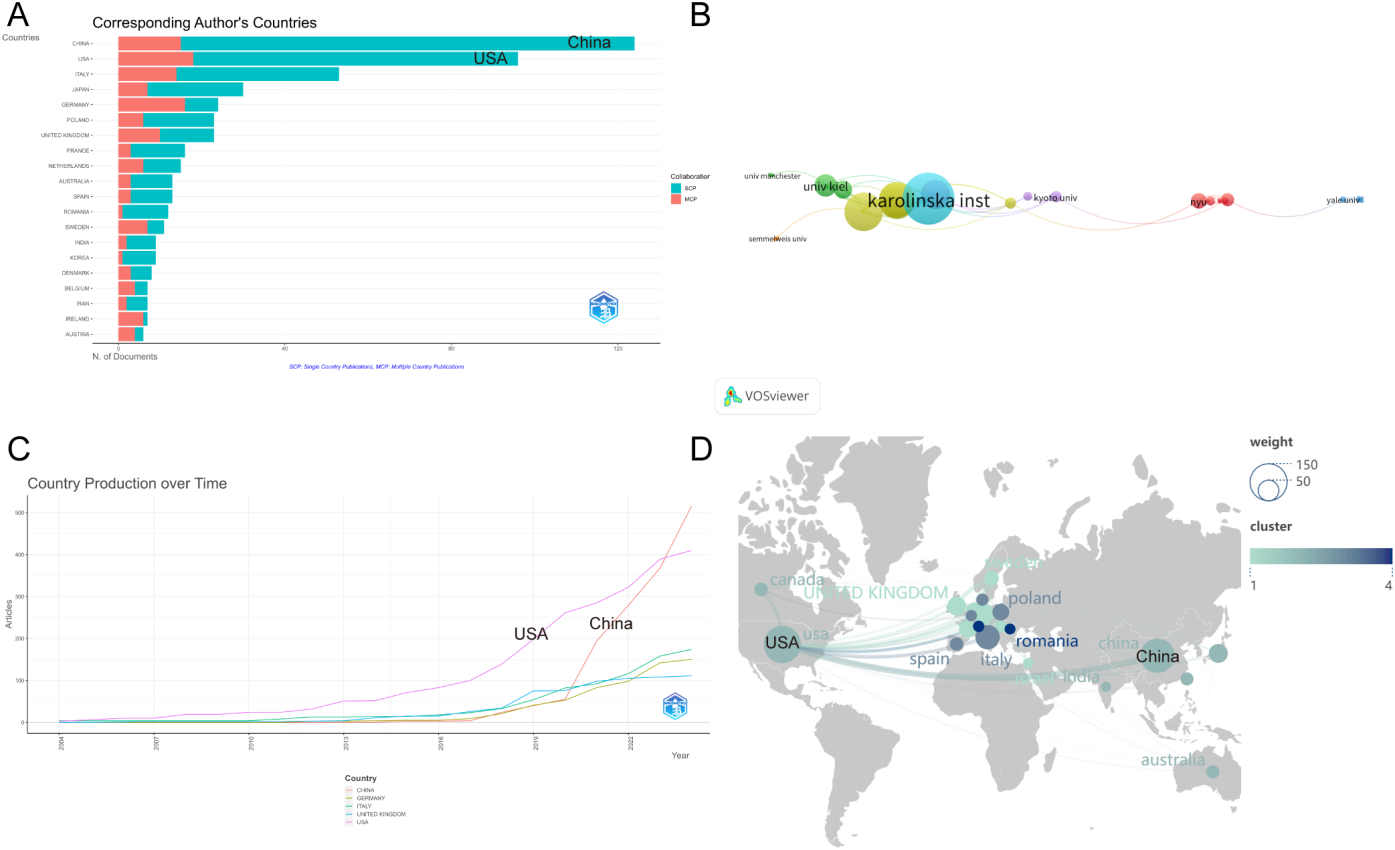
**(A)** Distribution of publications by author type across Top 10 Countries/Regions; **(B)** Temporal trends of psoriasis and gut microbiota research in Top 5 Countries; **(C)** Institutional collaboration network in psoriasis and gut microbiota research; **(D)** Geographic distribution and international collaboration of psoriasis research. From: VOSviewer.

In **Figure 2A**, the top 10 countries/regions are categorised based on the number of articles authored by a single corresponding author (in red) and multiple corresponding authors (in blue). **Figure 2B** shows the publication trends of the top five countries, illustrating how the number of publications on psoriasis and gut microbiota has changed over time. It highlights that China began to experience a rapid increase in publication volume starting in 2020 and has now become the leading country in terms of publication output, underscoring China’s current dominant position in psoriasis and gut microbiota genomics research.

**Figure 2C** illustrates the institutional collaboration network, where nodes with similar colors represent a common cluster, highlighting close collaborative relationships. The strength of the connecting lines between nodes indicates the intensity of cooperation between institutions. Karolinska Institutet stands out in the field and has the highest level of collaboration strength. It is the most closely connected university, having established collaborative relationships with 23 institutions. The results also show frequent exchanges between Karolinska Institutet and the University of Pécs, as well as with King’s College London.

**Figure 2D** depicts a map of publications by country/region. Nodes in different colors represent different countries/regions, with the size of each node reflecting the number of articles contributed by that particular country/region. The strength of the connections between nodes indicates the intensity of cooperation between countries.

**Supplementary Table 2** lists the top 10 authors in psoriasis and gut microbiota research based on their h-index. **Supplementary Figure 1** shows the top 10 authors by publication volume. A higher h-index indicates greater influence in the research field. Notably, four of the top authors are from Poland. **Supplementary Figure 2** identifies the authors with the highest total citation counts in the field. RUDNICKA LIDIA from the Medical University of Warsaw is not only the most productive author in this field, but also holds the highest h-index, g-index, and m-index. The authors with the highest total citation counts are RENDON ADRIANA (USA) and SCHAEKEL KNUT (Germany). Although their h-indexes are lower due to fewer publications, they are still core authors in the field of psoriasis and gut microbiota research. **Supplementary Figure 3** highlights BLASER, MARTIN J (USA, Rutgers University) as the most cited author in psoriasis and gut microbiota research. His papers have been cited the most by other studies in this field, indicating that his work has received widespread attention and recognition in this specific area of research.

**Figure 3:**
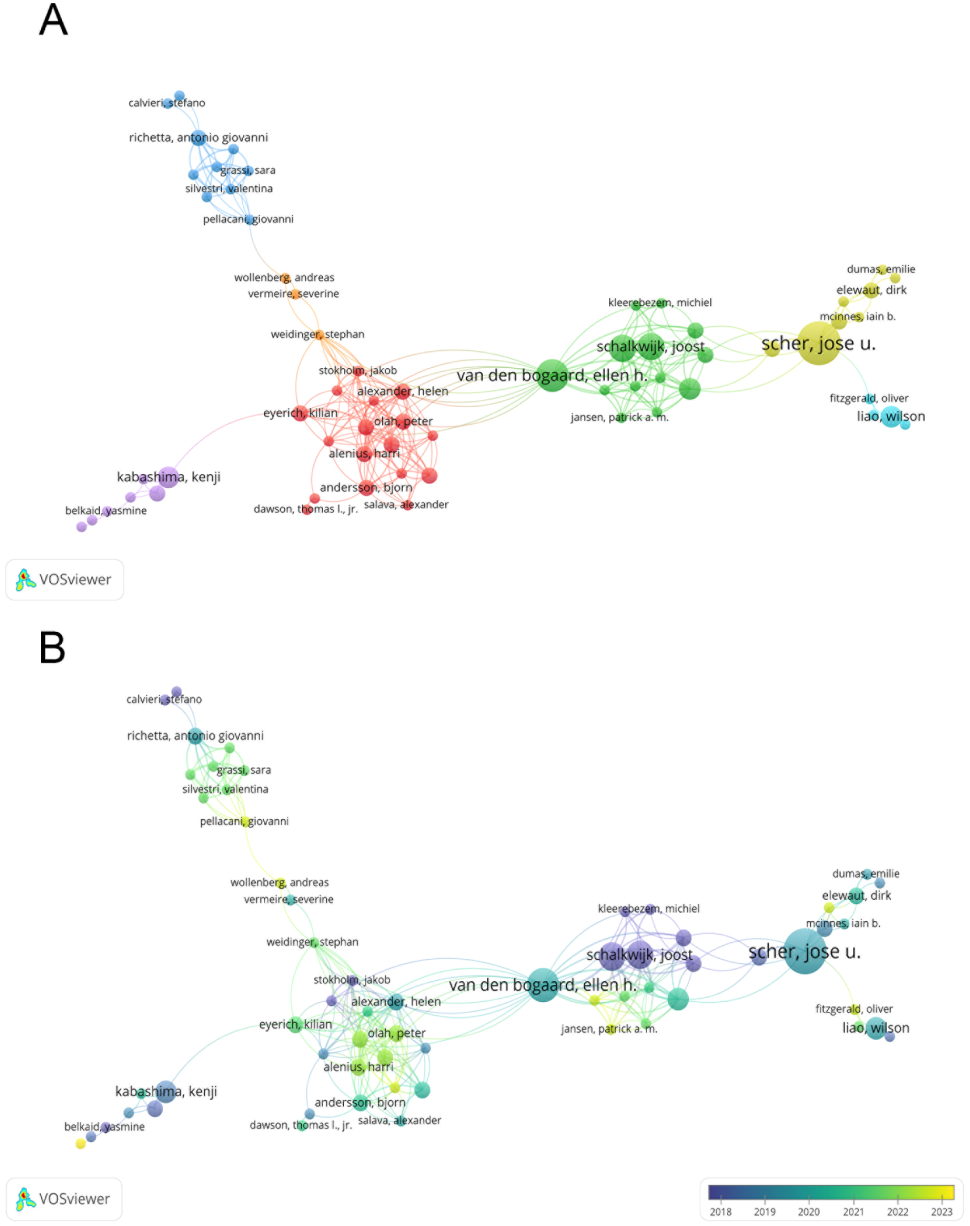
**(A)** Co-authorship network in psoriasis and gut microbiota research; **(B)** Temporal evolution of co-authorship in psoriasis and gut microbiota research. From: VOSviewer.

**Figure 3A** depicts a co-authorship network, illustrating the collaboration among authors in this field. **Figure 3B** adds a temporal dimension to this network, showing how author collaboration has evolved over time.

**Figure 4:**
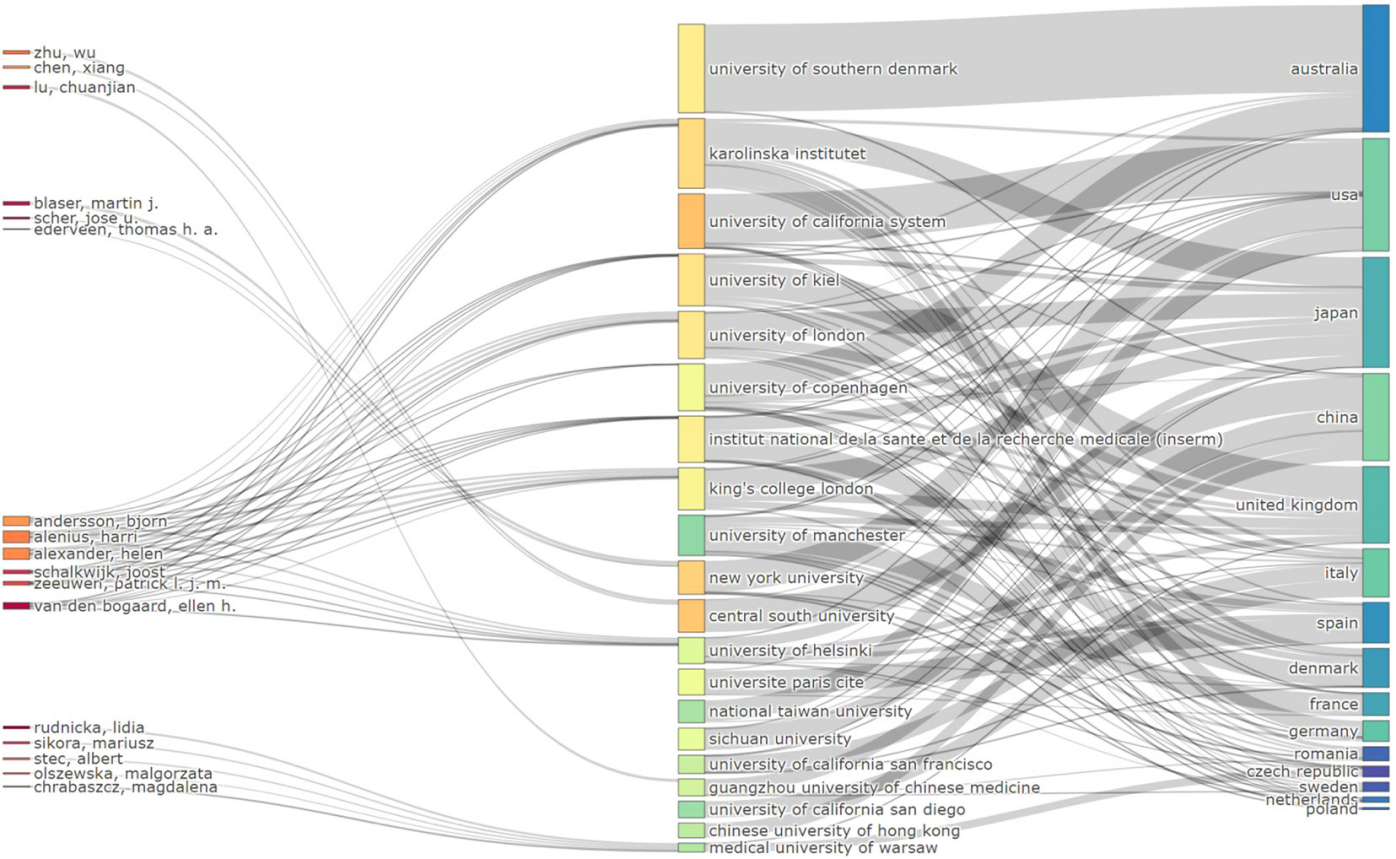
Sankey diagram of author-keyword-nationality connections in psoriasis and gut microbiota research.

**Figure 4** displays the connections between authors (left), keywords (center), and author nationality (right), with the width of the branches representing the volume of publications. This visualisation provides a comprehensive overview of the global research landscape and emphasises the central role of specific keywords and regions in shaping the field.

Through a detailed statistical analysis of academic publications, co-citations, and author collaboration, we gain a more accurate understanding of the knowledge landscape in psoriasis and gut microbiota. This analysis contributes to identifying the factors that have facilitated success in high-impact regions and institutions, providing valuable insights for further research.

### 3.3 Distribution of journals, co-cited journals, and co-cited references

Analyzing journal co-citations allows us to identify journals with significant influence in a specific research field. These studies were published in 254 different journals. The top 10 journals were selected based on the number of citations. The journals were ranked by total citations, H-index, and impact factor (2023). The most frequently cited journal is the *International Journal of Molecular Sciences*, which published 28 articles, accounting for 4.76% of the total, with a total of 1,664 citations. This journal also holds the highest H-index, G-index, and M-index. More than half of the top journals are Swiss journals. *Frontiers in Microbiology* ranks second in M-index.

**Supplementary Figure 4** presents the top ten most cited journals. *Journal of Investigative Dermatology* (J Invest Dermatol), *British Journal of Dermatology* (Brit J Dermatol), and *Journal of Allergy and Clinical Immunology* (J Allergy Clin Immunol) have each been cited more than 1,000 times, with *J Invest Dermatol* leading the list with a total of 1,643 citations. All of the top 10 journals have an impact factor (IF) greater than 3.5, with the *British Journal of Dermatology* having the highest IF (11). This journal was the first to publish research on psoriasis and gut microbiota and is one of the leading journals in dermatology, underscoring the significance and high quality of research in the field of psoriasis and gut microbiota. **Supplementary Figure 5** illustrates the journal co-occurrence network over time. It shows that in recent years, the most frequently published journals in the field are the *International Journal of Molecular Sciences* and *Frontiers in Microbiology*.

**Table 1** summarises the ten most locally cited references. The article *“ Decreased bacterial diversity characterises the altered gut microbiota in patients with psoriatic arthritis, resembling dysbiosis in inflammatory bowel disease”* by SCHER JU and colleagues, published in *Arthritis & Rheumatology* in 2015, is the most cited reference in the field, with 124 citations. The study explored the role of gut microbiota in psoriatic arthritis, finding that patients with psoriatic arthritis exhibit reduced gut microbiota diversity, similar to the dysbiosis observed in inflammatory bowel disease. These findings support the potential link between gut microbiota and systemic inflammation.

**Table 1:** Top 10 most local cited documents.

Rank	Title	Journal	DOI	Year	Local Citations	Normalised Local Citations
1	Decreased bacterial diversity characterizes the altered gut microbiota in patients with psoriatic arthritis, resembling dysbiosis in inflammatory bowel disease (Scher et al., 2015)	ARTHRITIS RHEUMATOL	10.1002/art.38892	2015	124	8.27
2	Community differentiation of the cutaneous microbiota in psoriasis (Alekseyenko et al., 2013)	MICROBIOME	10.1186/2049-2618-1-31	2013	102	3.29
3	Substantial alterations of the cutaneous bacterial biota in psoriatic lesions (Gao et al., 2008)	PLOS ONE	10.1371/journal.pone.0002719	2008	94	2.56
4	Comparison of bacterial microbiota in skin biopsies from normal and psoriatic skin (Fahlén et al., 2012)	ARCH DERMATOL RES	10.1007/s00403-011-1189-x	2012	80	7.41
5	Gut microbiota dysbiosis in a cohort of patients with psoriasis (Hidalgo-Cantabrana et al., 2019)	BRIT J DERMATOL	10.1111/bjd.17931	2019	78	6.58
6	Gut microbial composition in patients with psoriasis (Codoñer et al., 2018)	SCI REP-UK	10.1038/s41598-018-22125-y	2018	76	5.72
7	The Akkermansia muciniphila is a gut microbiota signature in psoriasis (Tan et al., 2018)	EXP DERMATOL	10.1111/exd.13463	2018	71	5.35
8	Alteration of the cutaneous microbiome in psoriasis and potential role in Th17 polarization (Chang et al., 2018)	MICROBIOME	10.1186/s40168-018-0533-1	2018	69	5.19
9	Psoriatic patients have a distinct structural and functional fecal microbiota compared with controls (Shapiro et al., 2019)	J DERMATOL	10.1111/1346-8138.14933	2019	68	5.74
10	Intestinal Microbiota Promotes Psoriasis-Like Skin Inflammation by Enhancing Th17 Response (Zákostelská et al., 2016)	PLOS ONE	10.1371/journal.pone.0159539	2016	63	6.53

The ten most cited papers globally are listed in **Table 2**. The article by RENDON A. and colleagues, published in 2019 in the *International Journal of Molecular Sciences*, is the most cited, with 1,048 citations. This paper discusses the close relationship between gut microbiota and inflammatory diseases in psoriasis patients. The study demonstrates that dysbiosis may exacerbate the inflammatory response in psoriasis by influencing the regulatory functions of the immune system. Changes in gut microbiota can directly affect the immune response, thereby intensifying systemic inflammation, particularly in diseases like psoriasis that have multi-system involvement. The article also explores the potential of modulating gut microbiota as a therapeutic target to improve clinical symptoms in psoriasis patients.

**Table 2:** Top 10 most globally cited documents.

Title	DOI	Total Citations	TC per Year	Normalized TC
Psoriasis Pathogenesis and Treatment (Rendon and Schäkel, 2019)	10.3390/ijms20061475	1048	174.7	14.9
Decreased bacterial diversity characterizes the altered gut microbiota in patients with psoriatic arthritis, resembling dysbiosis in inflammatory bowel disease (Scher et al., 2015)	10.1002/art.38892	575	57.5	5.0
The Gut Microbiome as a Major Regulator of the Gut-Skin Axis (Salem et al., 2018)	10.3389/fmicb.2018.01459	339	48.4	5.5
The Th17 pathway and inflammatory diseases of the intestines, lungs, and skin (Weaver et al., 2013)	10.1146/annurev-pathol-011110-130318	339	28.3	1.8
Cesarean section and chronic immune disorders (Sevelsted et al., 2015)	10.1542/peds.2014-0596	332	33.2	2.9
Substantial alterations of the cutaneous bacterial biota in psoriatic lesions (Gao et al., 2008)	10.1371/journal.pone.0002719	319	18.8	2.3
Bifidobacterium infantis 35624 modulates host inflammatory processes beyond the gut (Groeger et al., 2013)	10.4161/gmic.25487	308	25.7	1.7
Community differentiation of the cutaneous microbiota in psoriasis (Alekseyenko et al., 2013)	10.1186/2049-2618-1-31	275	22.9	1.5
The epithelial immune microenvironment (EIME) in atopic dermatitis and psoriasis (Dainichi et al., 2018)	10.1038/s41590-018-0256-2	248	35.4	4.0
Triggering psoriasis: the role of infections and medications (Fry and Baker, 2007)	10.1016/j.clindermatol.2007.08.015	242	13.4	1.8

**Figure 5:**
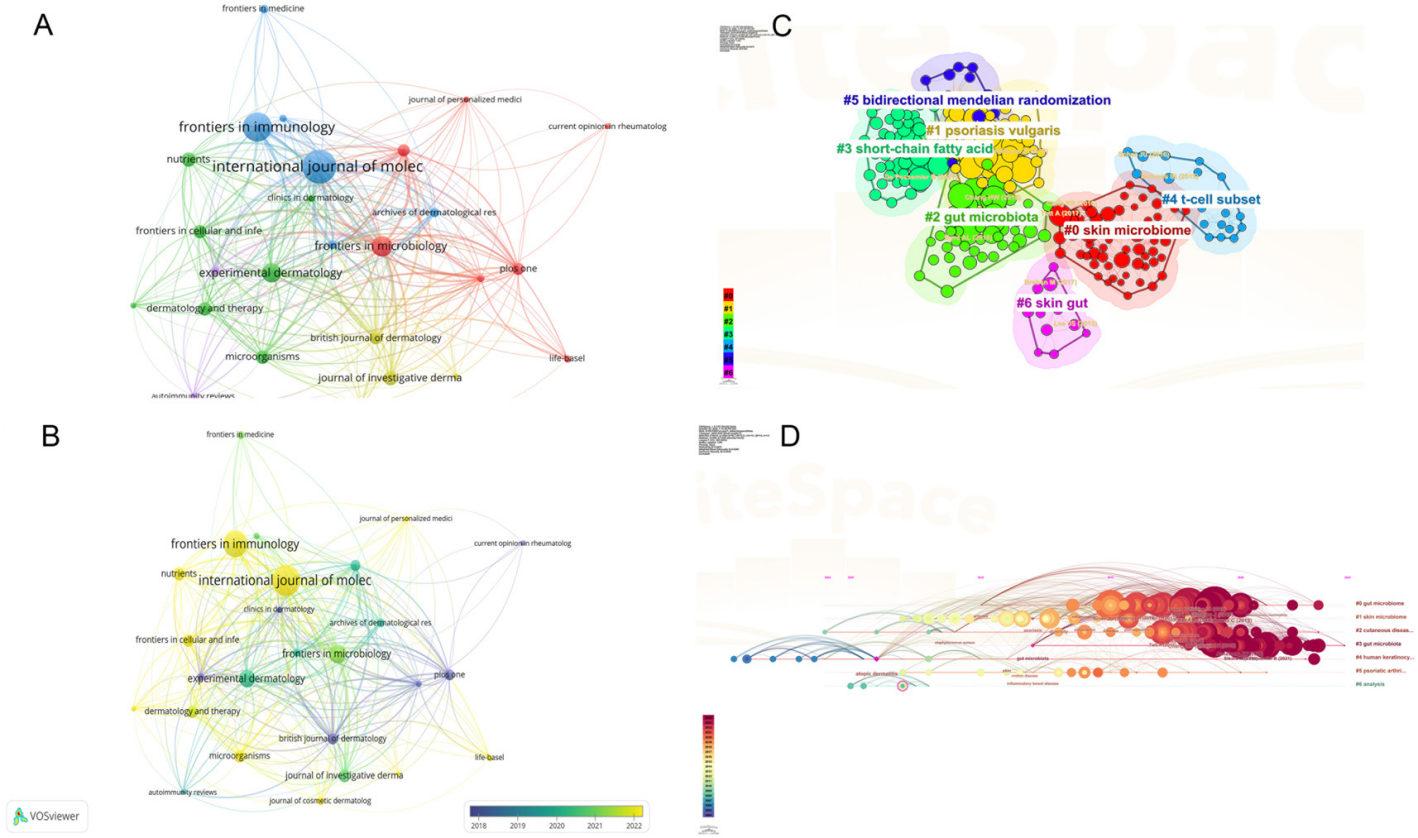
**(A)** Co-citation network of key references in psoriasis and gut microbiota research; **(B)** Top 100 most-cited articles in psoriasis and gut microbiota research; **(C)** Thematic clusters of co-cited references in psoriasis and gut microbiota research; **(D)** Temporal evolution of co-cited reference clusters in psoriasis and gut microbiota research. From: VOSviewer, CiteSpace.

**Figure 5A** illustrates the co-citation network of the literature, while **Figure 5B** presents the top 100 articles based on citation frequency. **Figure 5C** provides a visual network of co-cited references clustered by thematic direction, including terms such as *skin microbiome*, *plaque psoriasis*, *gut microbiota*, *short-chain fatty acids*, *T-cell subsets*, *bidirectional Mendelian randomization*, and *skin-gut axis*. **Figure 5D** displays a timeline of co-cited reference clusters from the past decade, allowing for an in-depth understanding of the evolution of the psoriasis-gut microbiota knowledge base. The initial cluster, labeled #0 *Skin Microbiome*, represents the embryonic stage of psoriasis microbiome research. This marks the beginning of targeted broad studies in the field, notably published by Elizabeth A. Grice in *Science*. Her study revealed significant diversity in skin microbiota across different body sites, with distinct differences between moist, dry, and sebaceous-rich areas. The skin microbiome shows both stability and variability in healthy versus disease states (e.g., psoriasis). Clusters #1 and #2 illustrate the current state of research in key areas. In cluster #3, an article by Xue Zhou (2024) in *Microbiology Spectrum* concisely discusses the role of *Eubacterium rectale* as a key gut microbiota marker in psoriasis patients. Reducing butyrate-producing bacteria, such as *Eubacterium rectale*, may promote systemic inflammation in psoriasis patients. Cluster #4 features the latest contributions from Jose U. Scher and colleagues, emphasizing the role of gut microbiota in inflammatory arthritis, particularly how specific gut microorganisms (e.g., *Lactobacillus bifidus* and *Prevotella*) influence immune responses and impact the development of inflammation and damage in psoriatic arthritis. Cluster #5 highlights recent studies focusing on how genetic variations affect gut microbiota diversity and composition, revealing gene-environment interactions in metabolic, nutritional, and immune health. Cluster #6 examines the relationship between biologics and gut microbiota in psoriasis.

**Figure 6:**
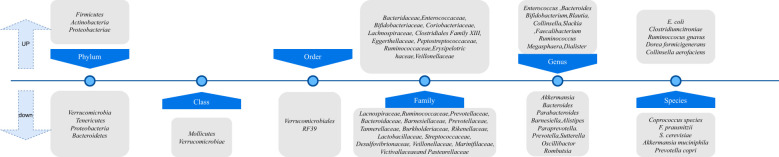
Taxonomic variation in gut microbiota between psoriasis/psoriatic arthritis patients and healthy controls.

Through the organization of key literature, **Figure 6** provides an overview of observational and interventional studies on the gut microbiota in patients with psoriasis and/or psoriatic arthritis, compared to healthy controls. The studies found that, compared to the healthy group, psoriasis patients exhibited a significant increase in gut microbiome diversity, with notable differences in the composition of phyla such as *Bacteroides* and *Firmicutes* (Codoñer et al., 2018; Todberg et al., 2021). Some studies have shown that regardless of genetic background, changes in the order *Clostridiales* and *Streptococcaceae* contribute to the exacerbation of disease progression in a mouse model of psoriasis induced by imiquimod (Zákostelská et al., 2016). These findings also support using symbiotic microorganisms as possible probiotic formulations.

### 3.4 Keyword analysis

**Figure 7:**
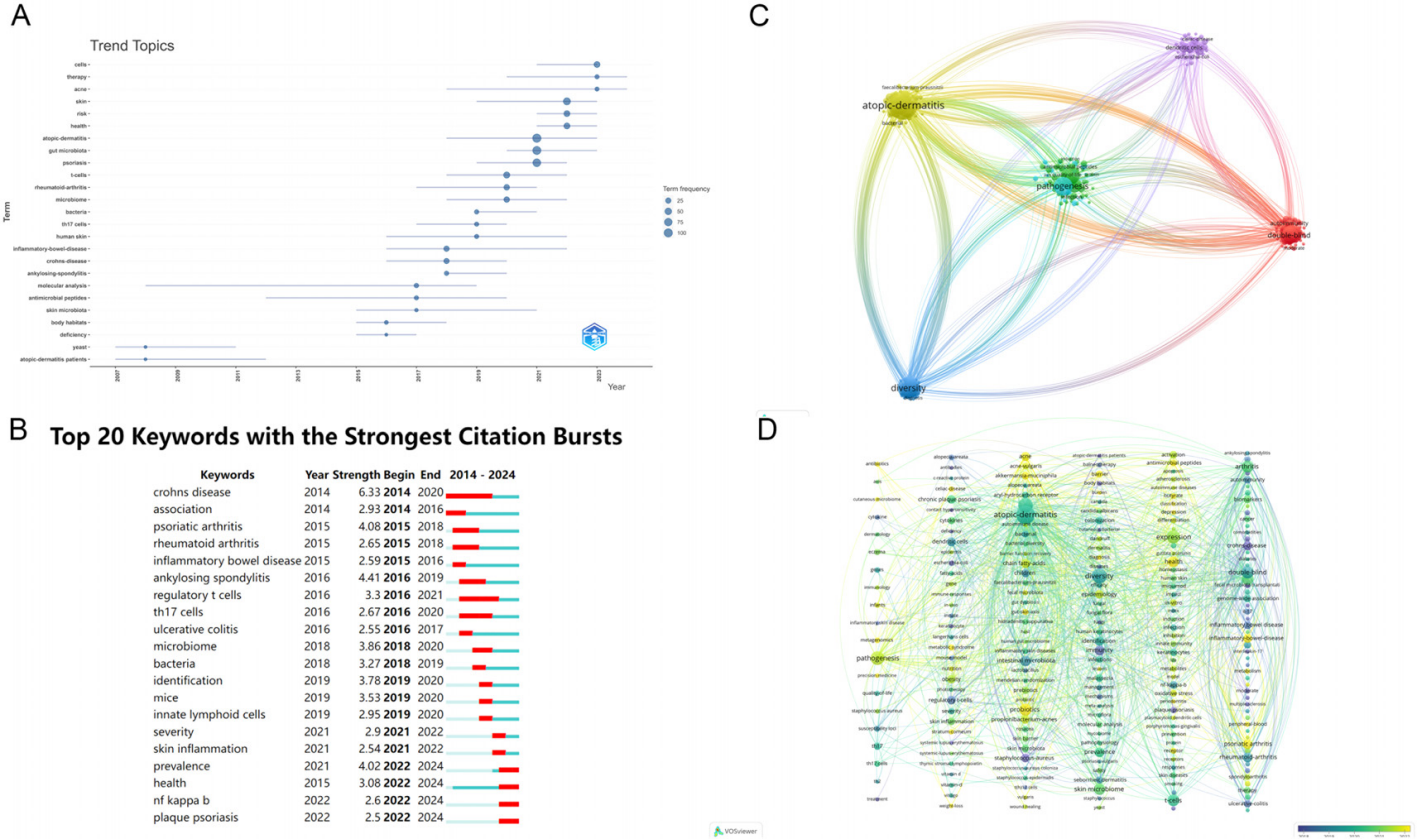
**(A)** Temporal trends of keywords in psoriasis and gut microbiota research; **(B)** Top 20 keywords and their temporal distribution in psoriasis and gut microbiota research; **(C)** Keyword co-occurrence network in psoriasis and gut microbiota research; **(D)** Clustered timeline of evolving research areas in psoriasis and gut microbiota research. From: VOSviewer.

After excluding redundant or irrelevant keywords, **Figure 7A** presents a temporal analysis of keyword trends. In **Figure 7B**, the top 20 keywords are displayed over time, with red dots indicating periods of high research activity, reflecting specific temporal hotspots in the field. This study identifies 20 influential terms related to the psoriasis drug delivery system, including *Crohn’s disease*, *association*, *psoriatic arthritis*, *rheumatoid arthritis*, *inflammatory bowel disease*, *ankylosing* sp*ondylitis*, *regulatory T cells*, *Th17 cells*, *ulcerative colitis*, *microbiome*, *bacteria*, *identification*, *mice*, *innate lymphoid cells*, *severity*, *skin inflammation*, *prevalence*, *health*, *NF-kappa B*, and *plaque psoriasis*. **Figure 7C** illustrates the keyword co-occurrence network in the research domain. In contrast, **Figure 7C** visualizes clusters of research within the field of psoriasis gut microbiome into five distinct areas: *Cluster #0 Local Transdermal Drug Delivery*, *Cluster #1 Atopic Dermatitis*, *Cluster #2 Dendritic Cells*, *Cluster #3 Pathogenesis*, *Cluster #4 Diversity*, and *Cluster #5 Double-Blind Studies*. To enhance clarity, **Figure 7E** integrates a timeline analysis on top of the clusters, offering a clearer view of how trends evolve. This approach also allows a better understanding of inter-cluster relationships through node linkages. The timeline analysis indicates that the current research frontiers include *Cluster #0 Precision Medicine*, *Cluster #1 Stratum Corneum*, *Cluster #2 Gut-Skin Axis*, *Cluster #3 Barrier Function*, and *Cluster #4 Antimicrobial Peptides in Inflammatory Bowel Disease*.

## 4 Discussion

This study is the first to apply bibliometric methods to analyze the research landscape of gut microbiota in psoriasis. Our findings reveal that gut microbiota and its metabolites have garnered significant attention in psoriasis research. 588 articles published between 2004 and October 15, 2024, were analyzed using data from the Web of Science (WOS) database.

### 4.1 Overview of the development of psoriasis and gut microbiota research

Based on the number of indexed publications, we identified two distinct phases in the growth of psoriasis and gut microbiota research. From 2004 to 2014, the annual number of publications was in the single digits, when high-throughput sequencing had not yet gained widespread adoption. During this time, most researchers relied on traditional culture-based methods. However, traditional microbial culturing techniques are inherently limited by the unavoidable enrichment or depletion of specific strains, which alters the original microbial ecosystem and can lead to significant bias in research findings.

The development of research literature is also closely linked to the strategic initiatives undertaken by various countries in gut microbiota research. Worldwide, numerous countries have launched national programs aimed at advancing microbiome research. For instance, the European Union led the “MetaHIT” (Human Microbiome Project) initiative in 2008, while the United States launched the “Human Microbiome Project” (HMP) in 2007. In 2017, China initiated its national program, the “Chinese Gut Metagenomics Project.” (Wang et al., 2020).

With these programs’ initiation, the research volume saw a significant increase after 2014. With the advent of high-throughput sequencing and big data technologies, scientists could perform bulk and single-cell sequencing of the gut microbiota, enabling rapid analysis of the microbial composition in the gut and its correlation with the health status of the subjects. The development of new technologies, the introduction and popularization of metabolomics, and the concept of the microbiome-skin axis have propelled the research on gut microbiota into a period of rapid growth. As government and international agency-supported initiatives were implemented, the number of studies reached its peak in 2021.

The number of publications from 2021 to 2024 has remained stable, indicating that research on the gut microbiota in psoriasis has entered a relatively mature phase. During this period, the changes in the gut microbiota in psoriasis have been thoroughly explored, with no significant technological breakthroughs. The pace of new research findings has stabilized. A significant reason for this stability is the lack of substantial changes in funding for this field.

Recently, a group of Chinese scientists, collaborating with researchers from 14 other countries including the United States, published a joint editorial in *Cell Research*, advocating for the initiation of Phase II of the Human Genome Project (HGP2). This article reflects the academic community’s recognition of gut microbiota research’s growing importance and direction. The study of gut microbiota in psoriasis is emerging as a hot research topic for the future. Since our statistics only cover up to October 2024, the number of publications in 2024 may further increase due to continued funding in the field and the promotion of metagenomic technologies.

However, research on the gut microbiota in psoriasis remains imbalanced, with China and the United States leading the field. The United States surpasses most countries in citation frequency, with the highest-cited authors and most productive institutions, demonstrating its scientific strength in this area.

Although China has fewer citations than the U.S. and Italy, it excels in the number of publications. This could be attributed to the widespread and cost-effective use of gut microbiota sequencing technology in both countries, which encourages more research. China’s research output significantly increased around 2020, following the adoption of high-throughput sequencing technology.

The number of studies in China surged around 2020, coinciding with the widespread adoption of high-throughput sequencing technology. The introduction and popularization of metabolomics and the microbiome-skin axis, along with strengthened international collaboration, also contributed to increased research output in China. Additionally, during the COVID-19 pandemic, the relationship between the gut microbiota and the immune system and infection prevention and control became key areas of focus, leading to an increase in publications on gut microbiota research after the pandemic.

China’s research volume is growing, but its average citation count remains low, likely due to two factors: most publications are recent (post-2020), and regional, dietary, and ethnic factors may influence gut microbiota composition, prompting researchers to cite domestic data more often.

We identified the changing trends of hotspots and frontiers in psoriasis gut microbiota research by analysing keywords and cited references. In 2004, Ulpu Saarialho-Kere introduced the concept of the gut-skin axis, initiating the study of the relationship between skin diseases and the gut microbiome (Saarialho-Kere, 2004). Some researchers have observed in clinical practice that several inflammatory bowel diseases may be associated with skin lesions, suggesting a connection between the gut and the skin (Torres et al., 2013). The analysis of citation bursts reveals two distinct phases in the gut microbiome and psoriasis research. From 2013 to 2018, the literature primarily focused on the relationship between the gut microbiome and inflammatory diseases such as psoriasis and rheumatoid arthritis. These studies highlighted changes in the diversity of the gut microbiome, particularly the reduction of certain bacterial species (e.g., butyrate-producing bacteria), which may lead to immune dysregulation and systemic inflammation. From 2019 onwards, research shifted towards more detailed mechanisms, exploring the interactions between specific microorganisms and host genes, as well as how the gut microbiome influences the pathogenesis of psoriasis through immune pathways. Studies by Chang HH (2017) and De Pessemier B (2021), among others, discussed how host-microbe interactions regulate inflammatory responses. Collectively, these studies emphasize the close relationship between systemic inflammation in psoriasis and gut dysbiosis, with the latter exacerbating the disease’s inflammatory response. Recent research has increasingly focused on developing therapeutic strategies by modulating the gut microbiome.

### 4.2 The mechanism of gut microbiota in psoriasis pathogenesis

**Figure 8:**
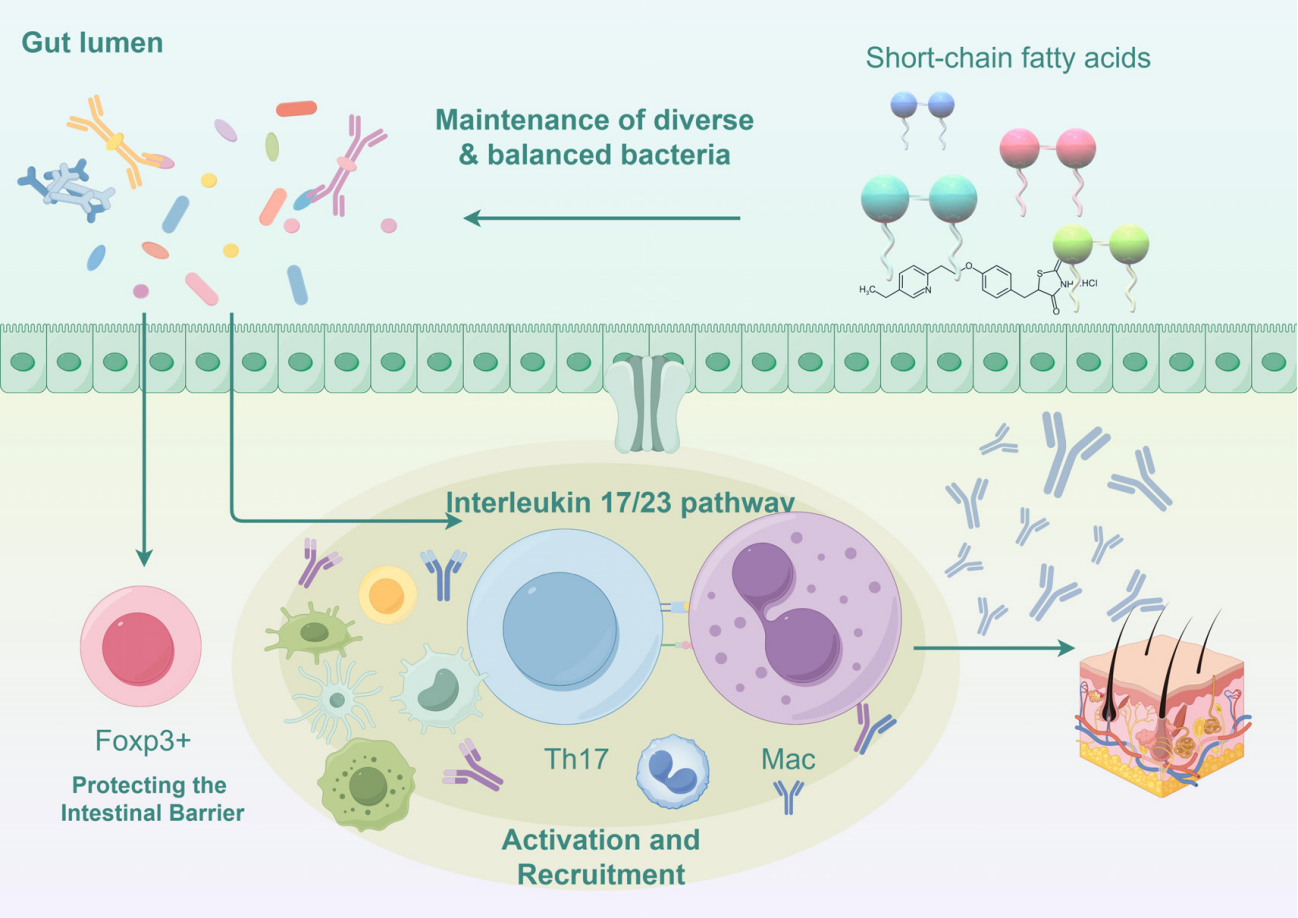
Mechanisms of gut microbiota influence on immune responses in psoriasis (Illustrated using Figdraw).

In our study, the *#3 pathogenesis* described in the clustering visualization analysis outlines the research on mechanisms. Through the keyword co-occurrence network, we identified a key mechanistic map showing how changes in the gut microbiota influence the immune response in psoriasis. (**Figure 8**).

#### 4.2.1 Disruption of the gut barrier function

The balance of the gut microbiota is crucial for maintaining the integrity of the intestinal barrier. Dysregulation of the ratio between *Firmicutes* and *Bacteroidetes* can affect carbohydrate metabolism and alter the production of short-chain fatty acids (SCFAs), leading to an increase in acetate production and a decrease in butyrate production (Chen et al., 2020; Shapiro et al., 2019). This imbalance ultimately results in chronic inflammation and damage to the intestinal epithelial barrier. This disruption facilitates the entry of pathogenic microorganisms and their metabolites into the bloodstream, where they can trigger systemic inflammatory responses by stimulating innate immune cells such as dendritic cells and macrophages. *F. prausnitzii* also affects gut barrier function by producing anti-inflammatory proteins, which are associated with the generation of intestinal epithelial cells (Effendi et al., 2022). These studies highlight the key concept of the microbiome’s involvement in maintaining a healthy gut barrier.

#### 4.2.2 Alterations in short-chain fatty acid metabolism

Certain beneficial bacteria in the gut microbiome (such as *Faecalibacterium prausnitzii* and *Eubacterium rectale*) can convert complex polysaccharides into SCFAs (such as butyrate). A decrease in SCFAs may impair the production of regulatory T cells (Tregs), which are controlled by the transcription factor Foxp3, thus reducing immune suppression and promoting the development of inflammatory diseases (Smith et al., 2013). The significant reduction in the abundance of *Akkermansia muciniphila* disrupts the pathway that converts mucin into SCFAs like acetate and propionate, leading to a decrease in antimicrobial peptides produced by host epithelial cells, which results in an imbalance in the body’s inflammatory responses (Scher et al., 2015; Widhiati et al., 2021). Additionally, the gut microbiota is essential for maintaining macrophage-dependent intestinal immune homeostasis mediated by SCFA-dependent pathways (Scott et al., 2018).

#### 4.2.3 Impaired function of regulatory T cells

Tregs are mentioned in the high-frequency keywords, and gut microbiome changes directly affect the generation and function of regulatory T cells. Faecalibacterium prausnitzii produces butyrate to maintain the Th17/Treg balance (Zhou et al., 2018). Certain beneficial gut bacteria, such as *Bifidobacterium*, can promote the differentiation of Tregs. However, a decrease in these microbial populations in psoriasis patients reduces Tregs (Dennis-Wall et al., 2017). Reduction in Tregs leads to excessive activation of pro-inflammatory immune cells (such as Th17 cells), exacerbating the inflammation in psoriasis (Olejniczak-Staruch et al., 2021). Metabolites produced by gut microbiota can interact with intestinal epithelial cells and immune cells, influencing the balance of Tregs and other immune cells, thereby regulating systemic immune responses.

#### 4.2.4 Excessive activation of Th17 cells and the IL-23/IL-17 pathway

The top 20 high-frequency keywords mention Th17 cells. A hallmark feature of psoriasis is the excessive activation of Th17 cells, which are significantly influenced by the gut microbiota. The severity of psoriasis correlates with increased Th17 cell activity (Zhao et al., 2023). Dysregulation of macrophage activity has consequences for T cell function, as certain pathogenic bacteria in the gut (such as *Prevotella* and *Ruminococcus*) adhere to intestinal epithelial cells and induce IL-17 production (Atarashi et al., 2015). IL-17 directly exacerbates skin inflammation by promoting keratinocyte proliferation and releasing inflammatory mediators. IL-23 plays a crucial role in maintaining Th17 cell activity, and gut dysbiosis further enhances Th17 cell activation, leading to worsening and refractory psoriasis (Stehlikova et al., 2019).

#### 4.2.5 Gut-skin axis imbalance

The timeline analysis of the main keywords *#2 gut-skin* axis shows the relationship between the gut and skin. The communication between the gut and skin involves the activation of immune components. Dysbiosis of the gut microbiota increases the secretion of inflammatory factors such as TNF-α and IL-6, which, upon entering the bloodstream, can further exacerbate skin inflammation (O’Neill et al., 2016; Thye et al., 2022). *Clostridium difficile* (formerly known as *Clostridium difficile*) produces metabolites such as para-cresol and phenol, biomarkers of gut dysbiosis. These metabolites have been shown to enter the bloodstream and accumulate in the skin, reducing skin hydration, impairing the integrity of the skin barrier, and affecting epidermal differentiation and keratinization (Dawson et al., 2011; Miyazaki et al., 2014). These inflammatory factors can also trigger inflammation in other organs, such as the joints, explaining the systemic immune dysregulation commonly seen in patients with psoriatic arthritis.

Fecal microbiota transplantation (FMT) directly alters the recipient’s microbiota, potentially restoring its normal composition. Various animal studies have validated that FMT transplantation can improve the symptoms in psoriasis mouse models.

### 4.3 Transformation of gut microbiota research into clinical application for psoriasis

In the timeline analysis, *#0 precision medicine* highlighted the therapeutic intervention potential of gut microbiota research. By summarizing the literature, we found that psoriasis patients’ gut microbiota changes are associated with the disease. Modulating gut microbiota could offer new therapeutic strategies for psoriasis (Widhiati et al., 2022; Zeng et al., 2024).

#### 4.3.1 Dietary intervention

Diet can directly intervene in the composition of the gut microbiota and the stability of the microbiota system (Wu et al., 2011). The Mediterranean diet has been confirmed to have anti-inflammatory and antioxidant effects (Flores-Balderas et al., 2023; Zou et al., 2024). Clinical studies have shown that adopting a Mediterranean diet can suppress the progression of psoriasis, regulate the balance of beneficial/harmful bacteria, and increase the diversity of gut microbiota in patients (Yoshida et al., 2023).

A high-fiber diet has also been proven to increase SCFA concentrations, reduce skin lesion thickness and inflammatory cell infiltration, and lower the expression of pro-inflammatory factors such as IL-17, thereby improving methotrexate-induced psoriasis.

#### 4.3.2 Fecal microbiota transplantation: evidence of efficacy

Fecal microbiota transplantation (FMT) directly alters the microbiota of the recipient, potentially restoring the normal composition of the microbiota. Fecal microbiota transplantation (FMT) significantly reduces the proportion of Th17 cells in the spleen of treated mice, increases anti-inflammatory IL-10 expression, and is closely associated with alleviating skin inflammation. However, clinical applications of FMT still need to consider issues such as infection risks and donor-recipient matching. Nevertheless, current research has confirmed its safety in psoriasis treatment (Flores-Balderas et al., 2023).

#### 4.3.4 Probiotic therapy and clinical trial results

Several studies have focused on the impact of *Bifidobacterium* species on skin diseases. For instance, *Bifidobacterium* CCFM683 has been shown to alleviate psoriasis in a dose-dependent manner by restoring the microbiota, regulating the FXR/NF-κB pathway, reducing pro-inflammatory cytokines, modulating keratinocytes, and maintaining epidermal barrier function (Miyazaki et al., 2014; Chen et al., 2023). A clinical randomized controlled trial involving 46 patients showed that probiotics supplementation significantly reduced markers of inflammation and oxidative stress, such as serum LPS and hs-CRP, thereby improving the patients’ quality of life (Moludi et al., 2022). Additionally, a meta-analysis of seven randomized controlled trials indicated that oral probiotics could improve psoriasis and reduce serum CRP levels, consistent with previous findings (Zhu et al., 2024).

## 5 Future research directions

Based on the current research, this study, using bibliometric methods and integrating relevant literature, presents the research process, research hotspots, and future directions regarding the involvement of gut microbiota in the pathogenesis and treatment of psoriasis (Widhiati et al., 2022; Zeng et al., 2024).

Prebiotics, probiotics, synbiotics, fecal microbiota transplantation, and dietary microbiota-targeted therapies have been shown to maintain the dynamic balance of the gut microbiota ecosystem through various pathways and mechanisms at a microscopic level, modulating the host immune system and alleviating psoriasis symptoms.

However, the connection between skin health and the immune response triggered by the gut microbiota remains largely unclear and requires further investigation (Wu and Wu, 2012; Lazar et al., 2018). Identifying the optimal probiotic or prebiotic candidates is crucial from a therapeutic perspective. Hotspot literature review indicates that *Akkermansia* and *Faecalibacterium* may be potential therapeutic targets for microbiota-related psoriasis. Yet, large-scale randomized controlled trials are still needed to validate this conclusion (Effendi et al., 2022). Therefore, there is an urgent need for large-scale research on the pathogenesis of psoriasis related to gut microbiota and the mechanisms of drug action. Understanding the functions of many poorly characterized microorganisms and expanding therapeutic options through microbiome interventions is vital, improving patients’ quality of life.

## 6 Conclusion

By conducting bibliometric analyses using software such as CiteSpace and VOSviewer, we have gained deeper insights into the development, hotspots, and future psoriasis and gut microbiota research trends over the past 20 years. The United States and China are leading countries in this field, underscoring the need for enhanced cooperation and communication among nations, institutions, and authors. This objective and quantitative approach provides researchers with important clues for understanding the structural and temporal dynamics of the discipline. Our analysis has identified shifts in research hotspots, and future research prospects may focus primarily on multi-omics data integration and bioinformatics to identify key microbial communities and metabolic pathways and implement microbial interventions. Additionally, strengthening international collaboration and data sharing will help improve the quality and impact of research, ultimately enhancing the prognosis and quality of life for patients with psoriasis.
