# Supplementary figures and images for: Mapping the global research landscape on psoriasis and the gut microbiota: visualization and bibliometric analysis
Source: Front Cell Infect Microbiol. 2025 Apr 25;15:1531355. doi: 10.3389/fcimb.2025.1531355 (PMC12062130; doi:10.3389/fcimb.2025.1531355)

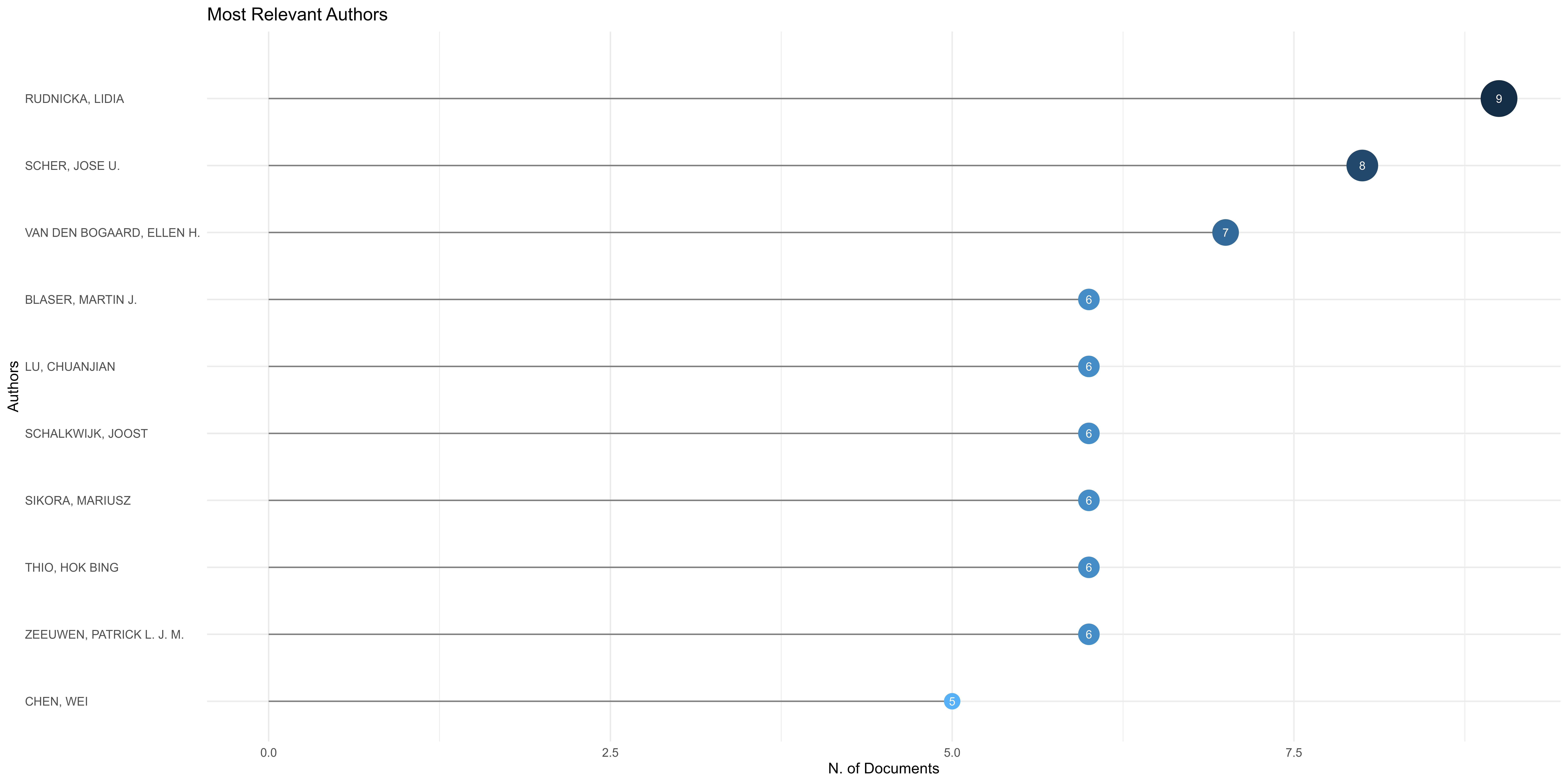

Supplement: Supplementary file 1 [file Image1.jpeg]

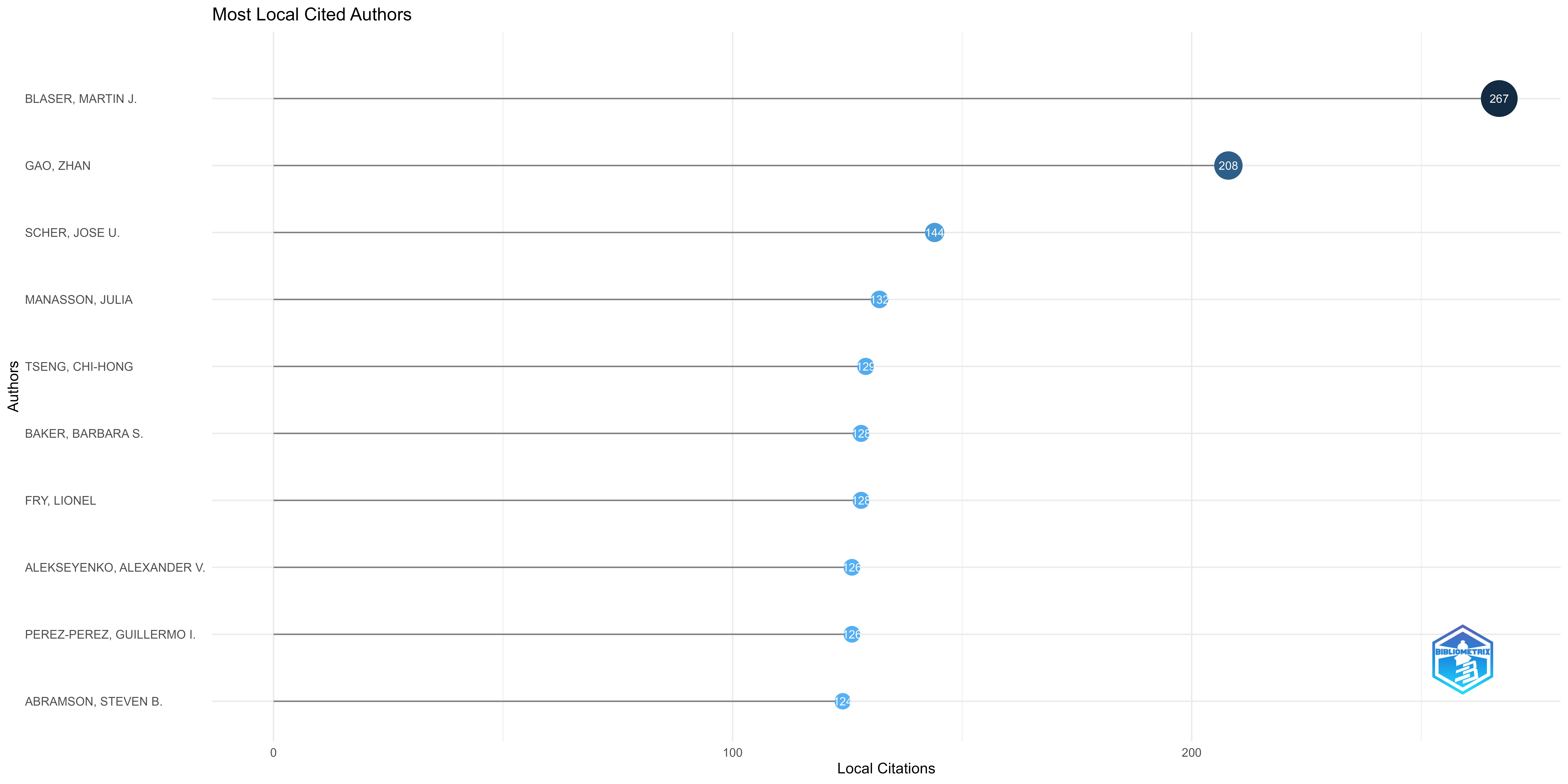

Supplement: Supplementary file 2 [file Image2.jpeg]

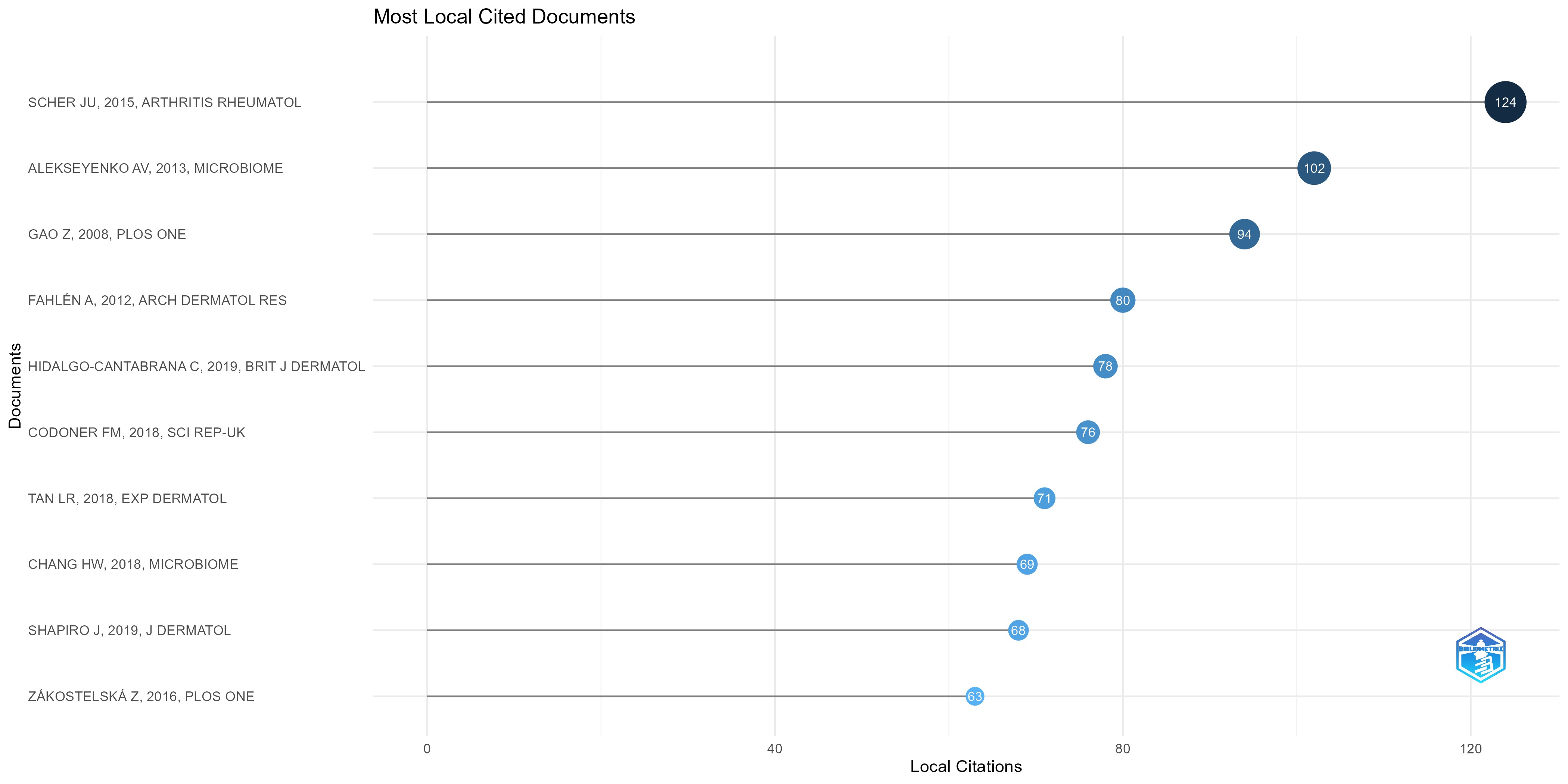

Supplement: Supplementary file 3 [file Image3.jpeg]

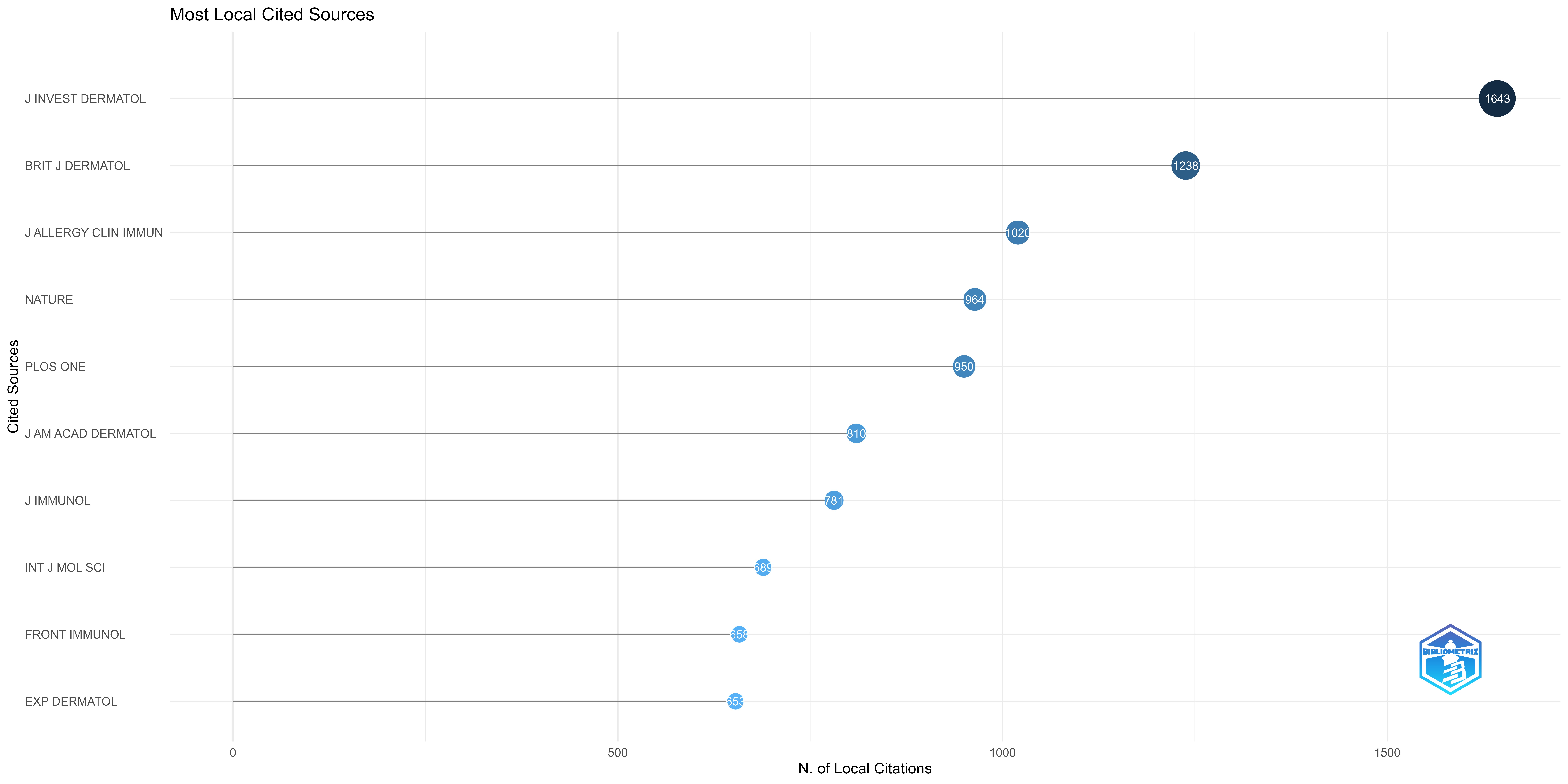

Supplement: Supplementary file 4 [file Image4.jpeg]

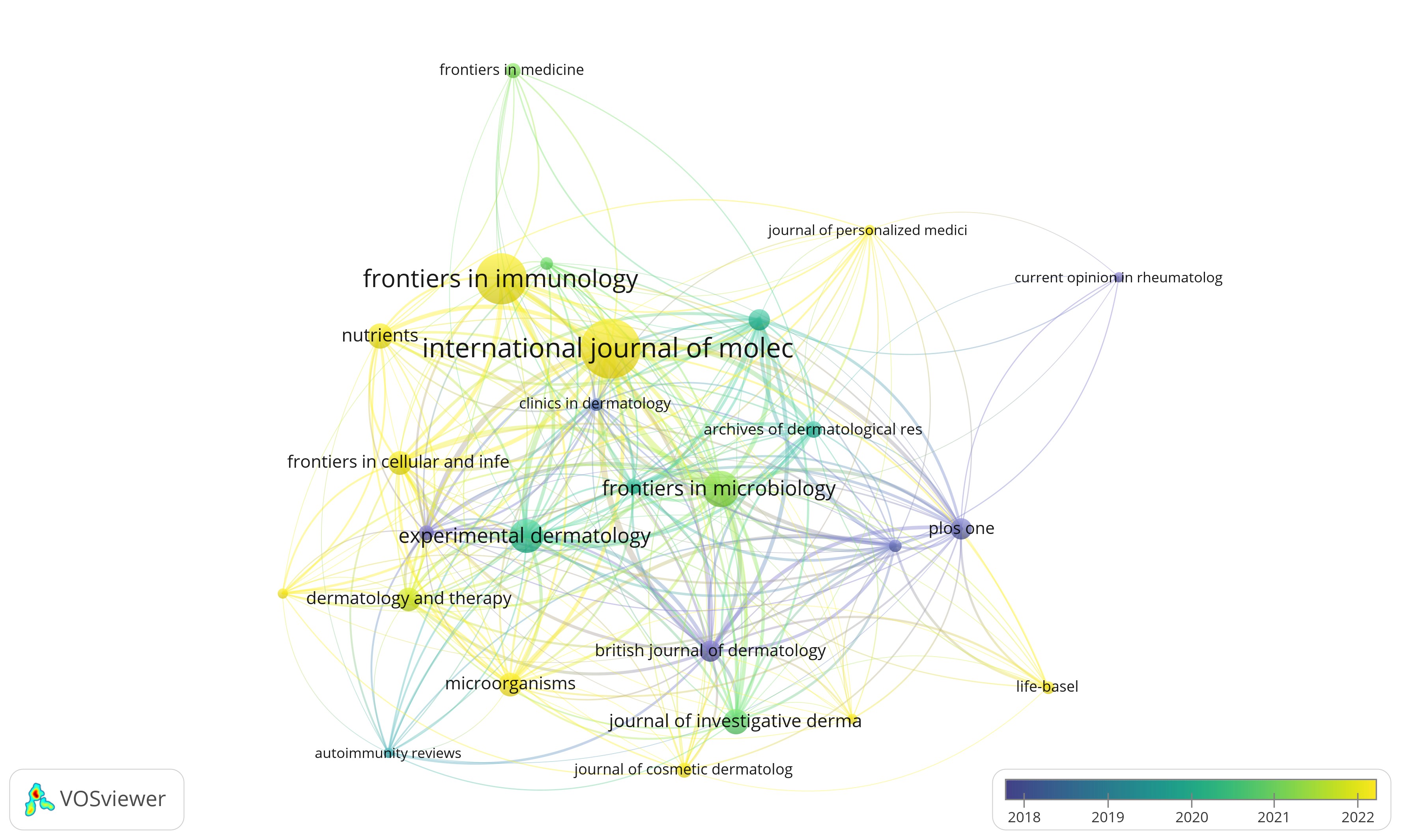

Supplement: Supplementary file 5 [file Image5.jpeg]
