# Supplementary material for: Mapping the global research landscape on psoriasis and the gut microbiota: visualization and bibliometric analysis
Source: Front Cell Infect Microbiol. 2025 Apr 25;15:1531355. doi: 10.3389/fcimb.2025.1531355 (PMC12062130; doi:10.3389/fcimb.2025.1531355)
Supplement: Supplementary file 6 [file Table1.docx]

| Supplement Table 1 top ten countries and institutions in terms of academic output | | |
| --- | --- | --- |
| Country | TC | Average Article Citations |
| USA | 5662 | 59.00 |
| GERMANY | 1781 | 74.20 |
| CHINA | 1681 | 13.60 |
| UNITED KINGDOM | 1337 | 58.10 |
| ITALY | 1086 | 20.50 |
| JAPAN | 986 | 32.90 |
| NETHERLANDS | 820 | 54.70 |
| SPAIN | 648 | 49.80 |
| BELGIUM | 586 | 83.70 |
| POLAND | 536 | 23.30 |
