# Supplementary material for: Mapping the global research landscape on psoriasis and the gut microbiota: visualization and bibliometric analysis
Source: Front Cell Infect Microbiol. 2025 Apr 25;15:1531355. doi: 10.3389/fcimb.2025.1531355 (PMC12062130; doi:10.3389/fcimb.2025.1531355)
Supplement: Supplementary file 7 [file Table2.docx]

| Supplementary Table 2 The Top 10 Authors Local Impact | | | | | | |
| --- | --- | --- | --- | --- | --- | --- |
| Author | h_index | g_index | m_index | TC | NP | PY_start |
| RUDNICKA LIDIA | 9 | 9 | 1.286 | 360 | 9 | 2018 |
| SCHER JOSE U. | 7 | 8 | 0.7 | 961 | 8 | 2015 |
| BLASER MARTIN J. | 6 | 6 | 0.316 | 1022 | 6 | 2006 |
| SIKORA MARIUSZ | 6 | 6 | 0.857 | 201 | 6 | 2018 |
| OLSZEWSKA MALGORZATA | 5 | 5 | 0.714 | 180 | 5 | 2018 |
| SCHALKWIJK JOOST | 5 | 6 | 0.385 | 554 | 6 | 2012 |
| STEC ALBERT | 5 | 5 | 0.833 | 155 | 5 | 2019 |
| VAN DEN BOGAARD ELLEN H. | 5 | 7 | 0.385 | 467 | 7 | 2012 |
| ZEEUWEN PATRICK L. J. M. | 5 | 6 | 0.385 | 554 | 6 | 2012 |
| ALEXANDER HELEN | 4 | 4 | 0.5 | 252 | 4 | 2017 |
